# A comprehensive review on the epidemiology of arboviruses in the Eastern Mediterranean Region (EMRO): insights from the WHO’s Regional Office 

**DOI:** 10.3205/dgkh000563

**Published:** 2025-06-27

**Authors:** Iman Owliaee, Mehran Khaledian, Ali Shojaeian, Farid Azizi Jalilian

**Affiliations:** 1 Department of Medical Virology, Faculty of Medicine, Hamadan University of Medical Sciences, Hamadan, Iran; 2 Department of Medical Entomology, Faculty of Medicine, Hamadan University of Medical Sciences, Hamadan, Iran; 3 Research Center for Molecular Medicine, Institute of Cancer, Avicenna Health Research Institute, Hamadan University of Medical Sciences, Hamadan, Iran

**Keywords:** arboviruses, arthropod-borne viruses, Crimean-Congo hemorrhagic fever virus, chikungunya virus, dengue virus, Rift Valley fever virus, West Nile virus, yellow fever virus, Zika virus, epidemiology, EMRO, Eastern Mediterranean countries

## Abstract

Arboviruses (arthropod-borne viruses) pose an ongoing public health threat in the Eastern Mediterranean Region (EMRO) of the World Health Organization. This review summarizes the epidemiology of major arboviruses – including Crimean-Congo hemorrhagic fever virus (CCHFV), chikungunya virus (CHIKV), dengue virus (DENV), Rift Valley fever virus (RVFV), West Nile virus (WNV), yellow fever virus (YFV), and Zika virus (ZIKV) in EMRO countries based on data from 2014–2023. Reported prevalence rates varied considerably between studies and countries, indicating localized transmission intensity. Overall, serological evidence confirms endemic circulation of CCHFV, CHIKV, DENV, and WNV in parts of the region. Large DENV outbreaks highlight it as a key concern. More systematic surveillance and standardized diagnostics are needed to characterize arbovirus epidemiology across the region and inform control strategies.

## Abstract (de)

Arboviren (arthropod-born viruses) sind eine ständige Bedrohung für die öffentliche Gesundheit in der Östlichen Mittelmeerregion (EMRO) der Weltgesundheitsorganisation. Diese Übersicht fasst die Epidemiologie der wichtigsten Arboviren – einschließlich des Krim-Kongo-Virus (CCHFV), des Chikungunya-Virus (CHIKV), des dengue-Virus (DENV), des Rift-Valley-Fieber-Virus (RVFV), des West-Nil-Virus (WNV), des Gelbfieber-Virus (YFV) und des Zika-Virus (ZIKV) – in den EMRO-Ländern auf der Grundlage von Daten aus den Jahren 2014-2023 zusammen. Die gemeldeten Prävalenzraten variierten je nach Studie und Land erheblich, was auf eine lokal begrenzte Übertragungsintensität hinweist. Insgesamt bestätigen die serologischen Nachweise die endemische Verbreitung von CCHFV, CHIKV, DENV und WNV in Teilen der Region. Große DENV-Ausbrüche machen deutlich, dass dieses Virus ein Hauptproblem darstellt. Eine systematischere Überwachung und standardisierte Diagnostik sind erforderlich, um die Arbovirus-Epidemiologie in der Region zu charakterisieren und Kontrollstrategien zu entwickeln.

## Introduction

### Virology

Arthropod-borne viruses, or arboviruses, constitute an ongoing and formidable risk to human and veterinary health [1], [2]. This diverse group of pathogens, which includes over 500 distinct viruses, is disseminated primarily through hematophagous arthropods, including mosquitoes, ticks, midges, and other blood-feeding insects, and through virus-infected vertebrate hosts [3]. Arboviruses are a major public health threat in the Eastern Mediterranean Region (EMRO) of the World Health Organization (WHO), which comprises 21 countries from Morocco to Pakistan [4], [5]. However, alternative transmission pathways within vertebrate populations have also been documented independently of arthropod vectors. These alternative pathways include mother-to-child transmission, nosocomial infections, subsequent transmission via blood transfusions or organ transplantation, and sexual transmission. Often viewed as incidental or secondary hosts in the viral life cycle, humans find themselves embroiled in the complex network of arboviral infections [6]. The past few decades have seen a rise in infectious diseases caused by arboviruses, leading to considerable loss of human and livestock lives and significant economic impacts [7]. Among this group of pathogens, several are implicated in severe diseases with global impact, and many have the potential to spark emerging infectious outbreaks. Examples of these virulent agents include dengue virus (DENV), chikungunya virus, West Nile virus (WNV), Japanese encephalitis virus (JEV), tick-borne encephalitis virus (TBEV), yellow fever virus (YFV), Rift Valley fever virus (RVFV), Sindbis virus (SINV), Crimean-Congo Hemorrhagic Fever virus (CCHF), and Zika virus (ZIKV) [8], [9]. 

The term “arbovirus” is a broad range of RNA viruses capable of infecting humans. This includes alphaviruses from the Togaviridae family, flaviviruses from the Flaviviridae family, bunyaviruses, nairoviruses, phleboviruses from the Bunyaviridae family, orbiviruses from the Reoviridae family, and vesiculoviruses [10]. Co-infections, especially those involving ZIKV and CHIKV, are widely reported and are thought to increase vector transmission and intensify ZIKV pathogenicity [10]. 

### Diagnostics

Differential diagnoses of such arboviral infections pose a complex challenge due to their often indistinguishable early-stage clinical symptoms. While molecular diagnostics are highly specific, they exhibit optimal sensitivity only during the acute phase of infection and can present logistical challenges in resource-limited endemic regions [7]. Several methods have been developed to detect the presence of these pathogens in clinical samples, including enzyme-linked immunosorbent assays (ELISAs), lateral flow assays (LFAs), and reverse transcriptase-polymerase chain reaction (RT-PCR) [11].

### Epidemiology

To date, 71 mosquito species have been recorded in Iran [12], [13]. Among these species, those belonging to the Culex and Aedes genera have emerged as the primary vectors of arboviruses [14]. In recent years, a surge in population movements, including refugee flows, has occurred, and there has been increased cross-border trade in goods and animals across regions like the EMRO. This region includes countries like Pakistan, which have experienced a significant rise in the burden of arboviral diseases [15], [16]. Understanding arboviruses is paramount in this context, given their profound impact on public health within the EMRO region. Arboviral diseases pose a substantial threat to the population in numerous EMRO countries. Through rigorous study, health authorities can gain insights into the epidemiology, transmission dynamics, and potential outbreak patterns of these viruses, thereby facilitating the development of effective prevention strategies and timely interventions to curtail the spread of arboviral disease [17]. Furthermore, examining arboviruses in the EMRO region is pivotal for developing diagnostic tools and treatment modalities. By elucidating specific viral strains and their genetic attributes, researchers can tailor targeted therapeutic approaches to combat these diseases with greater efficacy [18]. Additionally, studying arboviruses in this region enables the identification of potential reservoirs and vectors, a crucial step in implementing effective vector control measures and reducing the risk of future outbreaks [19]. Moreover, by sharing this knowledge with neighboring regions and international health organizations, EMRO can contribute significantly to global efforts to prevent the proliferation of arboviral diseases. 

This article provides an overview of the epidemiology of major arboviral diseases in EMRO countries, including their distribution, burden, outbreaks, and arthropod vectors. Understanding the epidemiological patterns and risk factors for arboviral transmission is critical for surveillance, preparedness, and response to mitigate the public health impact of these diseases in the region.

## Methods

A literature search was conducted in January 2024 to identify relevant studies on the epidemiology of major arboviruses in EMRO countries published during 2014–2023. To discover related studies, we conducted a comprehensive search of international databases such as PubMed, Scopus, Science Direct, Cochrane, Embase, Web of Science, and Google Scholar using combinations of the following keywords: arbovirus, Crimean-Congo hemorrhagic fever, CCHF, chikungunya, dengue, Rift Valley fever, West Nile, yellow fever, Zika, epidemiology, seroprevalence, EMRO, Eastern Mediterranean, Middle East, Africa, Asia. Reference lists of eligible articles were hand-searched for additional relevant studies. Original research articles published in English assessing arbovirus seroprevalence or viral detection in human populations in EMRO member states were included. Review articles, case reports, animal studies, studies from non-EMRO countries, and studies published before 2014 were excluded. The following information was extracted from eligible studies: first author, year of publication, country, sample size, participant characteristics, arbovirus(es) tested, diagnostic methods, and key seroprevalence or viral detection results.

## Results

### Crimean-Congo hemorrhagic fever (CCHF) 

#### Virology 

**Figure 1:**
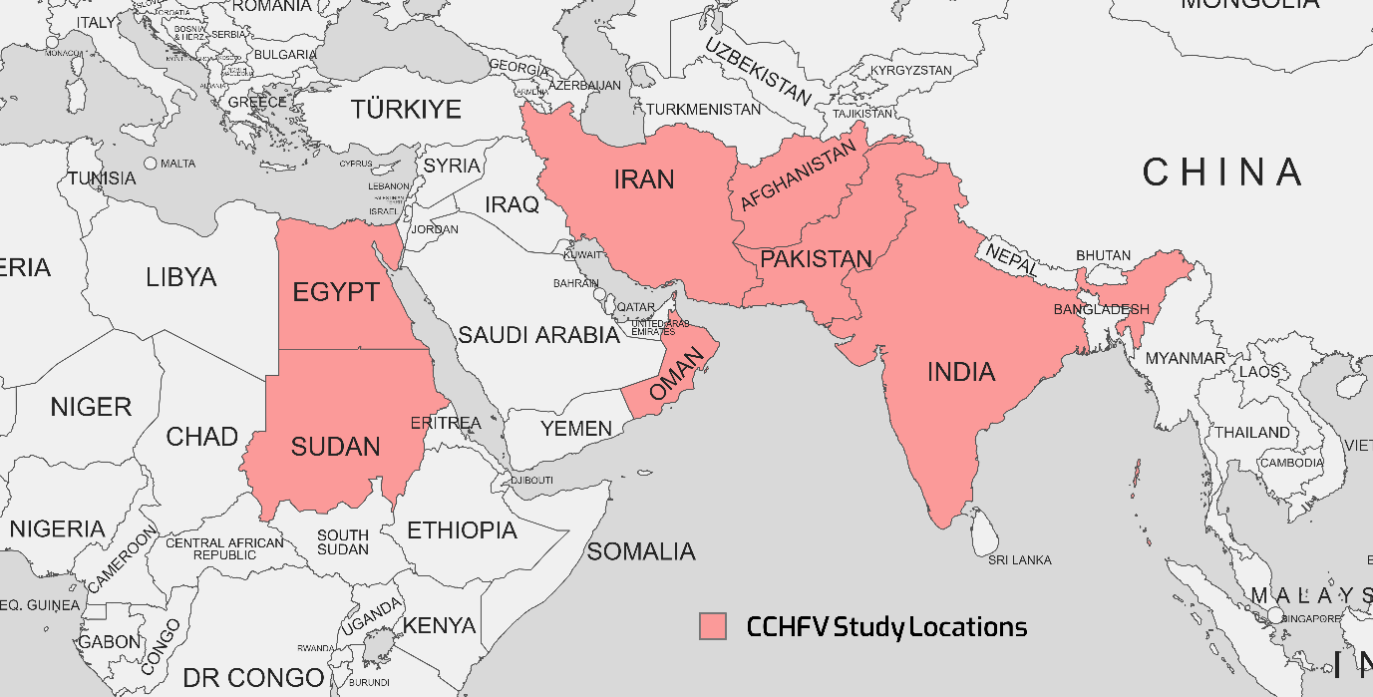
Geographical map of the distribution of CCHFV cases as reported in various studies

CCHF, a zoonotic viral hemorrhagic ailment, has established a substantial presence across diverse regions, including Asia, Africa, Eastern Europe, and the Middle East. CCHFV is classified under the Bunyaviridae family and the Nairovirus genus [20]. This organism's genome is made up of three segments: small (S), medium (M), and large (L). The L segment encodes the RNA-dependent RNA polymerase (RdRp), while the S and M segments encode the nucleocapsid and glycoprotein structural proteins, respectively [21]. The incidence of CCHFV-related diseases primarily afflicts individuals engaged in occupations such as ranching, farming, butchery, and slaughterhouses, with transmission occurring through occupational contact with infected animal blood and tissues, tick bites, and nosocomial infections [20], [22]. A diverse range of animals, including cattle, dromedaries, goats, sheep, and select reptiles and birds, can host the CCHFV. Ticks, notably Hyalomma marginatum and Hylomma rufipes, serve as both reservoirs and vectors for the virus [23]. Figure 1 (Fig. 1) illustrates the geographical map of the distribution of CCHFV cases.

#### Diagnostics 

The prevailing diagnostic method for detecting CCHFV in patient samples involves polymerase chain reaction (PCR) and reverse transcription-polymerase chain reaction (RT-PCR), a pivotal tool for virus detection. Rahden et al. [24], Sahak et al. [25], Shahbazi et al. [20], Todd et al. [26] and Khurshid et al. [27] used ELISA methods; Ahmed et al. [28], Habibzadeh et al. [22] and Umair et al. [21] used RT-PCR methods for detection of CCHF. 

#### Clinical aspects 

A majority of CCHFV infections remain subclinical or asymptomatic (approximately 90% of cases), while the fatality risk escalates to 10–40% when symptomatic manifestations occur. Common initial symptoms encompass headaches, myalgia, joint pain, elevated temperature, chills, diarrhea, nausea, and vomiting, often presenting mildly. A small minority of cases exhibit severe symptoms characterized by an abrupt onset, rapid onset of hemorrhaging, and significant hemorrhagic complications. The initial isolation of CCHFV from ticks in Kenya in 1975 marked a crucial milestone [20], [22], [23], [29]. The therapeutic options available to individuals afflicted with CCHF are primarily centered on supportive care, complemented by antiviral medication, notably Ribavirin [29]. 

#### Epidemiology 

Reports of CCHFV infections extend to EMRO nations, including Iran, Pakistan, India, Afghanistan, Sudan, and Oman. Table 1 (Tab. 1) summarizes seroprevalence studies detecting CCHFV antibodies or viral RNA published between 2015–2023 in countries endemic for the disease in the EMRO. Reported CCHFV seroprevalence ranged widely from 0.4% in Iran [20] to as high as 78% in Sudan [28]. Overall, countries in the region with the highest pooled CCHFV exposure were Afghanistan (mean 33.5%, range 4.1–50.7%) and Sudan (mean 20.7%, range 1.47–78%). Significant variability was noted even within the same country. This may be attributable to differences in geographic locales, seasons, climates, diagnostic methods, or study populations. Additional longitudinal investigations are warranted to clarify CCHFV epidemiology and risk factors for human infection across the Middle East, Africa, and Asia. Improved surveillance and control measures are needed to mitigate the morbidity and mortality caused by this neglected viral hemorrhagic fever. Both RT-PCR and ELISA demonstrated the ability to detect the virus. In some instances, RT-PCR identified more positive samples than paired ELISA testing, suggesting it may be a more sensitive diagnostic technique for this particular virus. However, ELISA may be more feasible in resource-limited settings.

### Chikungunya virus (CHIKV) 

#### Virology 

CHIKV, a member of the Togaviridae family in the alphavirus genus, is classified as an arbovirus due to its transmission by arthropod vectors. CHIKV is characterized by its enveloped nature, single-stranded positive-sense RNA genome, and affiliation with the flavivirus group. This virus primarily afflicts individuals residing in tropical and subtropical regions of Africa and Asia, mainly spreading through the *Aedes* species of mosquitoes, namely *Aedes aegypti* and *Aedes albopictus*, which serve as the vectors in this geographical context [15], [30]. As early as 1983, there were reports of CHIKV circulating among rodents in Pakistan, although human cases were few. In the midst of a dengue outbreak in Lahore in 2011, it was found that some patients also had antibodies to CHIKV. By 2016, CHIKV had surfaced in Karachi, and an outbreak was eventually declared once local transmission was confirmed [31].

#### Diagnostics 

The CHIKV can be diagnosed through various methods, such as virus isolation from clinical specimens such as serum or plasma by inoculation into susceptible cell lines (e.g., Vero, BHK-21); this is considered the gold standard, but it is time-consuming. Molecular methods like RT-PCR and real-time RT-PCR are suitable for rapid and sensitive detection of CHIKV RNA during the acute phase. Serological methods – such as ELISAs and rapid tests for detecting CHIKV-specific IgM and IgG antibodies in serum or plasma samples, and antigen detection using rapid diagnostic tests that detect CHIKV antigens in clinical specimens – are useful in resource-limited settings or outbreaks [32], [33].

#### Clinical aspects 

The initial clinical presentation of CHIKV infection often resembles other tropical fever illnesses. What sets CHIKV apart is the debilitating arthralgia it induces. This condition frequently affects multiple joints and exhibits a bilateral pattern. Additional clinical manifestations include conjunctivitis, asthenia, peripheral edema, headaches, and gastrointestinal disturbances. Moreover, CHIKV infection can lead to severe complications such as sepsis and the involvement of vital organs such as the heart, kidneys, and nervous system. Dermatological and ophthalmic manifestations are also not uncommon. Furthermore, CHIKV infection can have persistent consequences, including chronic pain, rheumatic symptoms, depression, and disturbances in mood and sleep patterns [34]. Without laboratory testing, cases of CHIK are often underreported because its signs and symptoms match those of dengue. Both diseases are transmitted by the same insect vectors [29]. 

#### Epidemiology 

**Figure 2:**
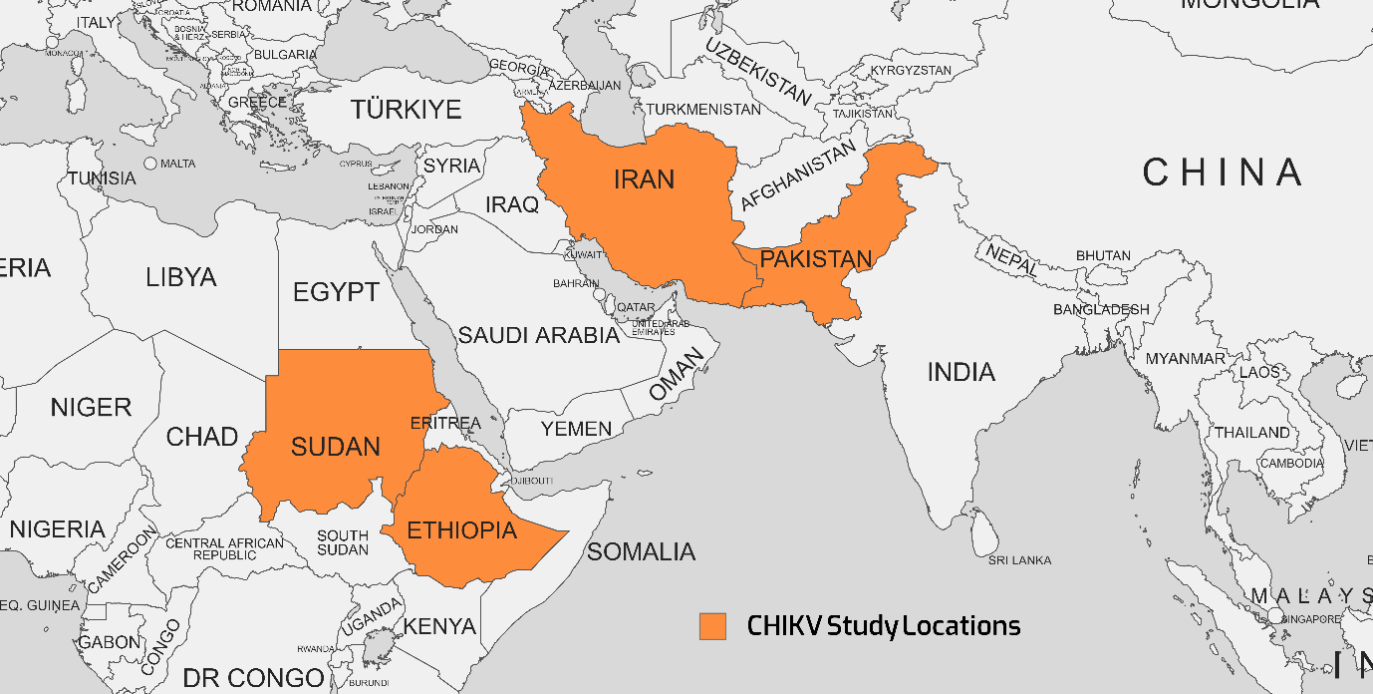
The geographical map of the distribution of CHIKV cases as reported in various studies

Historically, the first documented CHIKV outbreak occurred in 1952 on the Makonde Plateau, situated on the border between Mozambique and Tanzania [35]. Subsequently, the virus has reemerged in various regions, including Africa, Indian Ocean islands, South and Southeast Asia, the Americas, the Pacific, and Europe. This resurgence can be attributed, in part, to human migration across the Atlantic and Pacific oceans [36]. The geographical map of the distribution of CHIKV cases is shown in Figure 2 (Fig. 2).

Findings from studies between 2016–2021 that used RT-PCR and ELISA methods are presented in Table 2 (Tab. 2). Reported viral seroprevalence varied widely. In Pakistan, RT-PCR results ranged from 15.98% to 50% between 2018–2021. In Sudan, the seroprevalence of RT-PCR was as high as 84.5%, but as little as 0% in different study cohorts from 2019–2021. Both ELISA and RT-PCR detected the virus, but RT-PCR identified more positive cases in several studies, suggesting it may be a more sensitive test. The significant variability in viral prevalence, even within the same country and year, indicates localized epidemics and seasonal fluctuations rather than homogeneous endemic transmission. Herd immunity, circulating strains, geography, climate, social factors like crowding, and study sampling likely all contribute to the observed differences. Overall, the results confirm the circulation of this important virus in multiple Eastern Mediterranean nations in recent years. Ongoing surveillance and analysis of geographic and temporal patterns are essential to target public health interventions, e.g., vaccination, vector control, and behavior change communication, to the most affected populations. Improved reporting to regional databases would also help characterize the shifting epidemiology of this virus. Notably, Sudan reported both the highest and lowest seroprevalence rates. Furthermore, co-infection involving chikungunya and dengue viruses has been documented in two studies in Iran between 2021 and 2020, respectively [37], [38]. Detailed information regarding these co-infections is provided in Table 3 (Tab. 3), which separately discusses the epidemiology of the dengue virus.

### Dengue fever virus (DENV) 

#### Virology 

DENV is a mosquito-borne viral disease caused by one of four closely related dengue virus serotypes (DENV1–4) of the class flavivirus and family flaviviridae, which likewise incorporates chikungunya, yellow fever, and Zika viruses [39], [40]. Clinical manifestations of dengue infection vary, ranging from a self-limiting illness that resembles the flu to the severe lethal form of dengue hemorrhagic fever or dengue shock syndrome (DSS) [39]. *Aedes (A.) aegypti* and *A. albopictus* mosquitoes are the primary vectors of dengue in EMRO countries. Rapid urbanization and increased population density have promoted vector breeding in artificial containers. Climate factors also influence the spatial and seasonal distribution of vectors. Effective surveillance and targeted control of aedes mosquitoes are critical to prevent transmission [41]. The reservoir of this virus includes monkeys [42], and its vector is the *A. Aegypti* mosquito [39].

#### Diagnostics 

The diagnosis of DENV infection can be achieved through various methods, including molecular techniques such as RT-PCR and qRT-PCR, which are highly sensitive and specific for detecting DENV RNA during the acute phase of infection. Virus isolation through inoculation of clinical samples into mosquito cell lines or mammalian cell cultures is time-consuming and requires specialized facilities. Serological assays, such as ELISAs and rapid diagnostic tests (RDTs) for detecting DENV-specific antibodies (IgM and IgG) in serum or plasma samples, are useful in the later stages of infection. Antigen detection assays, including non-structural protein 1 (NS1) capture ELISAs and RDTs, can detect DENV antigens during the early phase of illness [43], [44].

#### Clinical aspects 

About 75% of dengue infections are asymptomatic, and 5% of cases are severe. If appropriately treated, the case:fatality ratio for severe dengue can be as low as 0.1% and as high as 10%. Both symptomatic and asymptomatic individuals can spread DENV to mosquitoes that bite them throughout the seven-day infectivity period, since they are both viremic. The intrinsic incubation period of DENV in humans, which is the time before symptoms appear, is 3–14 days [45]. 

#### Epidemiology 

**Figure 3:**
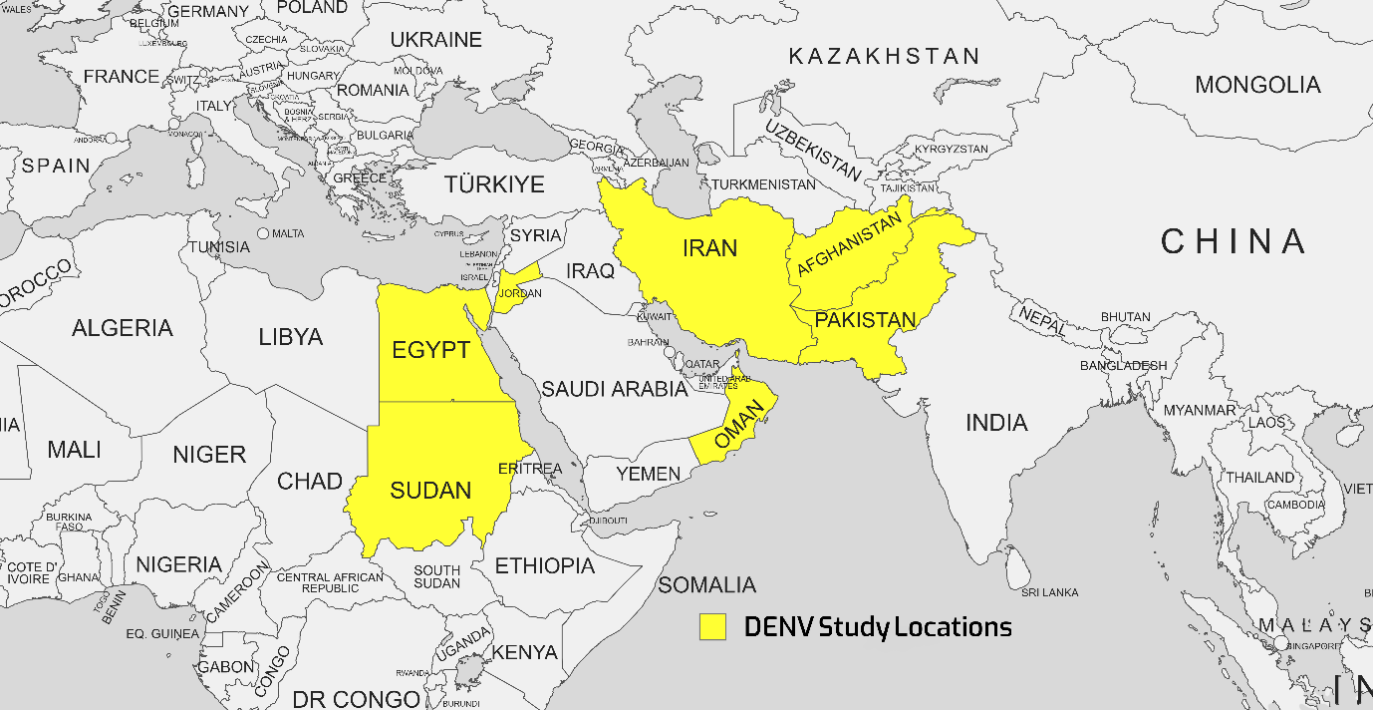
Geographical map of the DENV case distribution

Many variables, such as human population expansion, density, migration, cross-border commerce and travel, water shortages and inadequate water storage, as well as global climate change, might contribute to the spread of dengue [46]. Two of the most prevalent mosquito-borne diseases in Southeast Asia, the Western Pacific region, and the United States are DF and dengue hemorrhagic fever (DHF). Iran has always been vulnerable to DENV because of its geographic location and proximity to DENV-endemic countries such as Afghanistan and Pakistan [47]. Since 1799, there have been reports of dengue outbreaks in Egypt, specifically in the governorates of Cairo and Alexandria [48]. The lack of authorized dengue treatments or vaccinations puts the world's population at risk of illness during a pandemic [40]. Dengue is widespread in EMRO countries, with epidemic activity reported in most countries in recent years. It is estimated that 100 million dengue infections and over 20,000 deaths related to severe dengue occur in the region annually [49]. Figure 3 (Fig. 3) shows the geographical map of the DENV case distribution.

Table 3 (Tab. 3) summarizes over 30 studies on the seroprevalence of DENV in EMRO countries between 2014 and 2023. The main diagnostic methods used were ELISA serology testing and RT-PCR to detect viral RNA. The prevalence rates varied widely across the different studies and countries. Reported DENV seroprevalence varied widely, ranging from 2.5% to 100% in Pakistan [50]. 

In Pakistan, multiple studies over the years consistently found some of the highest DENV exposure, with positive rates reaching 69% by RT-PCR [51]. Serotypes DENV-2 and DENV-3 predominated. Sudan also exhibited high DENV seroprevalence, up to 70% in certain locales [46]. Other countries had more moderate exposure – around 17–26% in recent studies from Jordan, Egypt, and Oman. Afghanistan and Iran showed lower DENV seropositivity, less than 5% in the included investigations. Significant differences were observed between ELISA and PCR-based assessments, with PCR detecting acute infection and ELISA measuring historical exposure. Additional factors like geographic region, population, season, sample size, and epidemic years likely contributed to variability between studies. Overall, the table highlights DENV as an important public health threat in the Middle East and southern Asia. Improved surveillance, vector control, and vaccines are needed to prevent DENV epidemics and reduce the disease burden in endemic countries. In summary, the dengue virus remains an important public health threat with evidence of ongoing endemic transmission in parts of the EMRO. Continued surveillance and standardized methods are needed to elucidate the epidemiology fully.

### Rift Valley fever virus (RVF) 

#### Virology 

RVF, a mosquito-borne disease caused by the RVFV (genus Phlebovirus, family Bunyaviridae), has caused epidemics in Africa and, more recently, the Arabian Peninsula, that are affecting both livestock and humans [52]. The virus, transmissible through mosquito bites, exposure to infected animal fluids, or consumption of raw milk from infected animals, was documented outside Africa in Saudi Arabia and Yemen in 2000, potentially due to imported infected animals [53]. Additionally, human and vector mosquito populations can continue transmitting RVFV vertically [54]. Mosquitoes are the primary RVF vectors, especially Aedes and Culex species, which proliferate after heavy rains and floods [55].

#### Diagnostics 

DENV infection can be diagnosed using various methods, including nucleic acid amplification tests (NAATs) and IgM antibody testing. NAATs are the preferred method for patients with a clinically compatible illness who live in or recently traveled to a disease-endemic area. Laboratory confirmation can be made using RT-PCR or NS1 antigen. IgM detection is most useful for patients presenting more than one week after fever onset. Both ELISA and RT-PCR are approved as in vitro diagnostic tests [44], [56].

#### Clinical aspects 

The recent outbreak presented a febrile hemorrhagic syndrome with liver and renal dysfunction, resulting in 882 confirmed cases and 124 deaths (possibly underreported). Travelers to endemic areas risk acquiring RVF [97]. RVF in humans usually manifests as a mild illness with influenza-like symptoms. However, in 1–3% of patients, the disease can progress to a severe condition characterized by hemorrhaging, potentially fatal encephalitis, liver necrosis, ocular disease, both internal and external bleeding, and other symptoms [57]. Pregnancy-related infections pose a significant risk to both the mother and fetus. Such infections can lead to fetal deformity, miscarriage, or preterm delivery. 

#### Epidemiology 

Emerging vector-borne diseases caused by pathogens such as ZIKV, WNV, Japanese encephalitis virus, Venezuelan equine encephalitis virus, malaria, brucellosis, and dengue can result in similar complications [52]. In nations where RVF is endemic, especially Sudan, which relies on the export of animals and animal products, RVF outbreaks pose substantial public health and economic risks [54]. RVF was initially identified among exotic sheep between Lake Naivasha and Lake Elmenteita in the Kenyan Rift Valley in 1910 and 1912 [58]. Later, in 1944, Smithburn, who was employed by the Yellow Fever Research Institute in Entebbe, Uganda, obtained two virus isolates from mosquitoes [59]. These mosquitoes, belonging to the *Aedes tarsalis* and the *Eretmapodites* spp., were collected from the untamed forest of Western Uganda [58], [60]. The seroprevalence rates reported vary considerably between the studies (Table 4 (Tab. 4)). In 2020, Ahmed et al. [54] tested samples using RT-PCR and found an average seroprevalence of 33.3% with a wide range from 0.1% to 99.2%. This indicates substantial geographic variation in viral circulation within Sudan in that year. In contrast, two studies from 2019 found a much lower seroprevalence of 6.8% [9] and even 0% seroprevalence [61]. Other factors like seasonality, study population characteristics, sample size, and assay methods could also contribute to the variability. Overall, the results confirm that this virus circulates in Sudan, but prevalence appears to fluctuate dramatically based on setting and period. Improved surveillance and standardized diagnostics are needed to characterize the epidemiology of this virus in Sudan fully. Risk factors for hot spots of infection require further study to support evidence-based control measures. 

### West Nile virus (WNV) 

#### Virology 

WNV is a positive-sense, single-stranded RNA virus that is spread by mosquitoes and belongs to the family flaviviridae [62]. WNV is also the most frequently reported *Culex*-transmitted virus in Iran. Ornithophilic mosquitoes and migratory birds perpetuate the WNV enzootic cycle, but mammals (e.g., humans and horses) do not. Members of the genus *Culex (C.)*, primarily the *C. pipiens*, *C. univittatus, C. antennatus*, and *C. vishnui* complex, are the main mosquito vectors [16], [63]. Although the primary mode of WNV transmission to humans is through mosquito bites, it can also spread in several other ways. These include blood transfusion, breastfeeding, laboratory occupational exposure, and organ transplants [62], [64]. 

#### Diagnostics 

WNV diagnosis involves several methods. Clinicians typically consider WNV in patients with compatible symptoms who live in or recently traveled to an endemic area within two weeks before symptom onset. The preferred laboratory method is testing serum or cerebrospinal fluid (CSF) for WNV-specific IgM antibodies. These antibodies are usually detectable 3–8 days after symptom onset and persist for 30 to 90 days. However, cross-reactivity with other flaviviruses can complicate interpretation. IgG antibodies indicate previous infection, while plaque-reduction neutralization tests (PRNTs) can determine the specific infecting flavivirus. Molecular tests (viral cultures and RT-PCR) can confirm infection, but their likelihood of detecting WNV is low. Immunohistochemistry (IHC) detects WNV antigens in tissue samples. State public health laboratories or the CDC can perform these tests [65], [66].

#### Clinical aspects 

Most WNV infections are asymptomatic, but in 20% of instances, the infection causes West Nile Fever (WNF), and in fewer than 1% of cases, it causes acute West Nile Neuroinvasive Disease (WNND) [63]. In 80% of patients, the infection is asymptomatic. Without this, infected individuals may develop a severe febrile illness. The severity of this illness can range from a self-limited infection to encephalitis, which can result in long-term impairment and death in 10% of neurologic cases [67]. 

#### Epidemiology 

**Figure 4:**
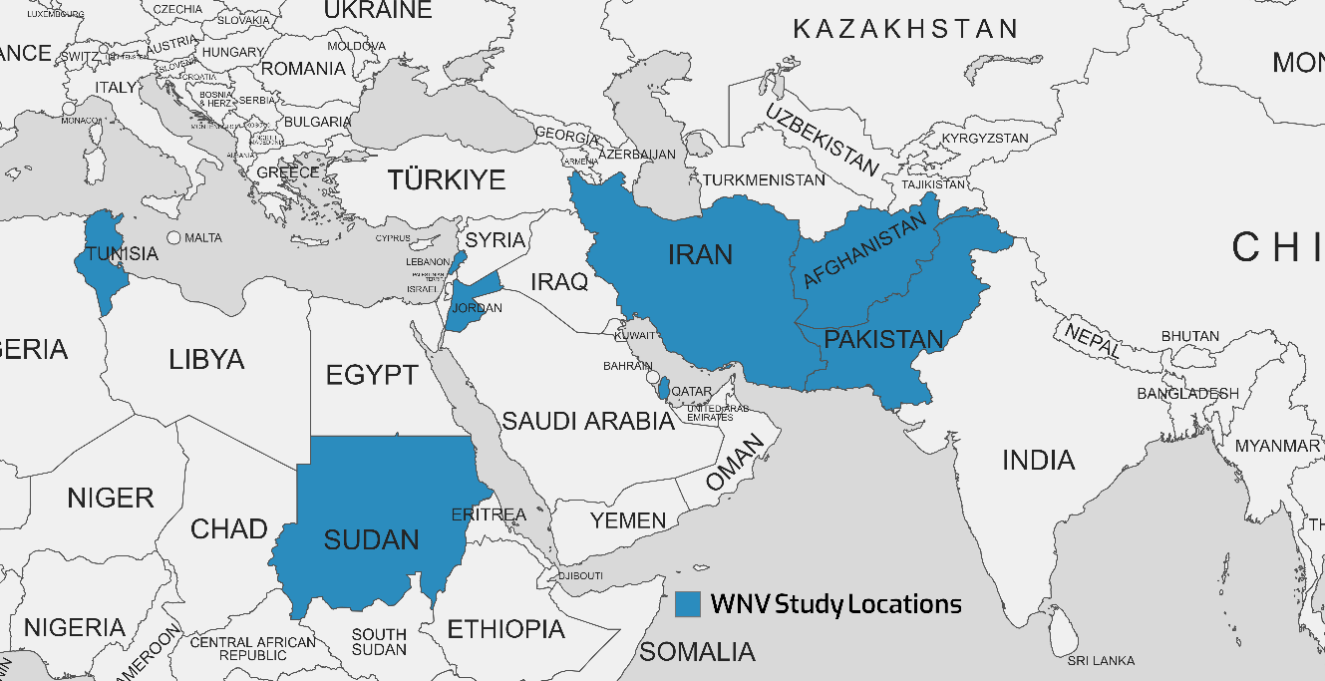
Geographical map of the distribution of WNV cases

WNV isolates from various geographical locations have been divided into up to nine lineages by phylogenetic analysis. Outbreaks of major viral encephalitis in humans have been associated with lineages 1, 2, and 5. WNV infections have been reported both in Pakistan and in neighboring countries such as Iran, China, and India [62]. The WNV, first discovered in 1937 in the West Nile region of Uganda, has since been linked to epidemics across Africa, the Middle East, especially Egypt, and Western Asia. Outbreaks have been reported in North America and Europe since the 1990s [64], [68]. Table 5 (Tab. 5) summarizes findings from 8 studies between 2014–2023 that investigated the seroprevalence of a specific virus in several Eastern Mediterranean countries, including Pakistan, Sudan, Iran, Jordan, Lebanon, and Qatar. Reported WNV seroprevalence ranged from 0.5% in Afghanistan [69] to 28.8% in Tunisia [70]; Figure 4 (Fig. 4) shows the geographical map of the WNV case distribution. In general, the highest WNV exposure was found in Tunisia, with around 10-30% positive rates. Lebanon, Jordan, Iran, and Pakistan followed with moderate WNV seroprevalence of 1.9–20.6% in recent studies. The lowest seropositivity was observed in Afghanistan and Qatar (0.5–3.3%). Significant differences were noted between PCR and ELISA results, since PCR detects current WNV infection while ELISA measures historical exposure. Geographic region, seasonality, study population, and sample size likely contributed to variability between studies.

Overall, Table 5 (Tab. 5) indicates that WNV remains an endemic public health concern in the region. Additional surveillance is warranted, especially in Tunisia, which has very high infection rates. Improved mosquito control and personal protective measures are needed to reduce WNV transmission to humans. WNV vaccination may benefit high-risk groups in endemic areas. Improved reporting to WHO and other regional databases would strengthen the evidence base for targeted public health interventions.

### Yellow Fever Virus (YFV) 

#### Virology 

YFV is a zoonotic flavivirus with a positive-sense, single-stranded RNA genome, which is prevalent in the tropics of South America and Africa. The *Haemagogus, Sabethes*, and *Aedes genera* of mosquitoes are the vectors responsible for maintaining and transmitting the YFV reservoir to humans. This process is known as the “sylvatic cycle” and often occurs when humans encroach upon the native habitats of monkeys. Once YFV has infected humans, it is disseminated through an “urban cycle” by a new vector, the anthropophilic *A. aegypti* mosquito [71], [72], [73]. 

#### Diagnostics 

YFV can be detected using various methods, including real-time RT-PCR during the initial viremic phase, virus isolation in cell cultures or inoculation in mice, serological assays like ELISA and IIF, and immunohistochemistry in fatal cases. Molecular techniques such as RT-PCR are used to detect viral RNA in blood samples, while isolating the virus requires biosafety l Level 2 containment. However, these methods can exhibit cross-reactivity with other flaviviruses. Immunohistochemistry can provide a definitive diagnosis in fatal cases. The PRNT is considered the gold standard for confirming YFV infection due to its high specificity [74], [75].

#### Clinical aspects 

The WHO defines a YF-suspected case as any person who exhibits an acute febrile fever and the development of jaundice within 14 days after the onset of symptoms [76]. Clinical manifestations range from a mild flu-like illness to hemorrhagic fever, with a possible case fatality rate of 20% to 50%. Usually, those who visit or work in jungle environments are at risk of unintentional exposure to the virus [72]. Previous studies have demonstrated the persistent presence of RNA from various flaviviruses, including YFV, dengue virus, and ZIKV, in urine or saliva [77]. 

#### Epidemiology 

**Figure 5:**
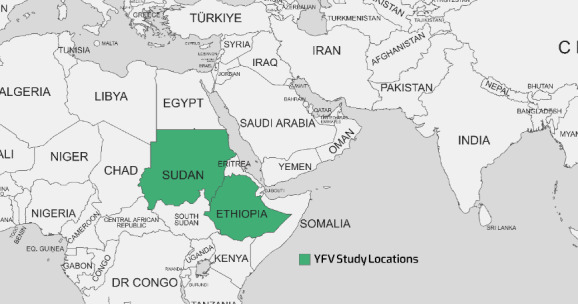
A geographical map of the distribution of YFV cases as reported in various studies

Nigeria, Uganda, Ghana, Chad, Guinea, the Republic of the Congo, and Angola are African countries that have recently experienced YF outbreaks. Since the 1960s, numerous YF outbreaks in Ethiopia have resulted in the deaths of over 30,000 people in the southern region. An YF outbreak re-emerged in the South Omo Zone of southern Ethiopia in 2013, leading to many fatalities [71], [76]. The seroprevalence rates varied considerably across the studies, ranging from 0% in 2019 to 46% in Sudan in 2016 [78]. The 0% seroprevalence found with RT-PCR testing in Sudan in 2019 indicates that the virus may have been absent or circulating at extremely low levels in the particular geographic area and period sampled. This contrasts sharply with the 46% seropositivity rate found just three years prior in Sudan in 2016, signaling an active epidemic at that time. The 2.9% seroprevalence found in Ethiopia in 2021 by Asebe et al. [79] suggests more limited transmission but still with evidence of circulation, as shown in Table 6 (Tab. 6). Figure 5 (Fig. 5) depicts the geographical map of the YFV case distribution.

### Zika virus (ZIKV) 

#### Virology 

ZIKV is an emerging pathogen of significant public health concern. While most infections result in self-limiting symptoms, the recent outbreak has been linked to an increased incidence of congenital anomalies, including microcephaly. ZIKV is primarily transmitted by *Aedes* mosquitoes, particularly *A. aegypti* and *A. albopictus*. Structurally, ZIKV belongs to the flavivirus family, sharing closer genetic ties with DENV and YFV and more distantly with WNV. Its genome consists of a single-stranded, positive-sense RNA encoding a polyprotein that undergoes cleavage into functional domains, including capsid, the precursor of the membrane (prM), and envelope (E), along with seven nonstructural proteins (NS1, NS2A, NS2B, NS3, NS4A, NS4B, and NS5) [80], [81]. 

#### Diagnostics 

Zika virus diagnosis involves two main methods: direct detection of viral components and antibody-based tests. NAATs, particularly RT-PCR, are the gold standard for confirming an active infection, targeting viral RNA in bodily fluids. Serological assays such as ELISA detect antibodies against ZIKV, which is useful in later stages or in identifying past exposure. However, RT-PCR offers the most definitive diagnosis during the initial stages of ZIKV infection, as it may show cross-reactivity with other flaviviruses [82], [83].

#### Clinical aspects

Clinical symptoms of ZIKV infection include low-grade fever, maculopapular rash, arthralgia, and non-purulent conjunctivitis. Despite extensive research, no specific antiviral therapies or vaccines are available for ZIKV, making it a significant global health challenge. It can be passed from a mother to a fetus during pregnancy and can also be transmitted through sexual contact [84]. ZIKV can spread in several ways, including urine, mosquitoes bite, sexual intercourse, blood transfusions, and transmission from mother to fetus. The incubation period for ZIKV is around one week, after which it can cause disorders such as Guillain-Barré syndrome (GBS), microcephaly, and symptoms such as joint pain, skin rash, headache, fever, and conjunctivitis. Some people may also experience vomiting, diarrhea, eye redness, lethargy, and edema [85], [86]. 

#### Epidemiology 

**Figure 6:**
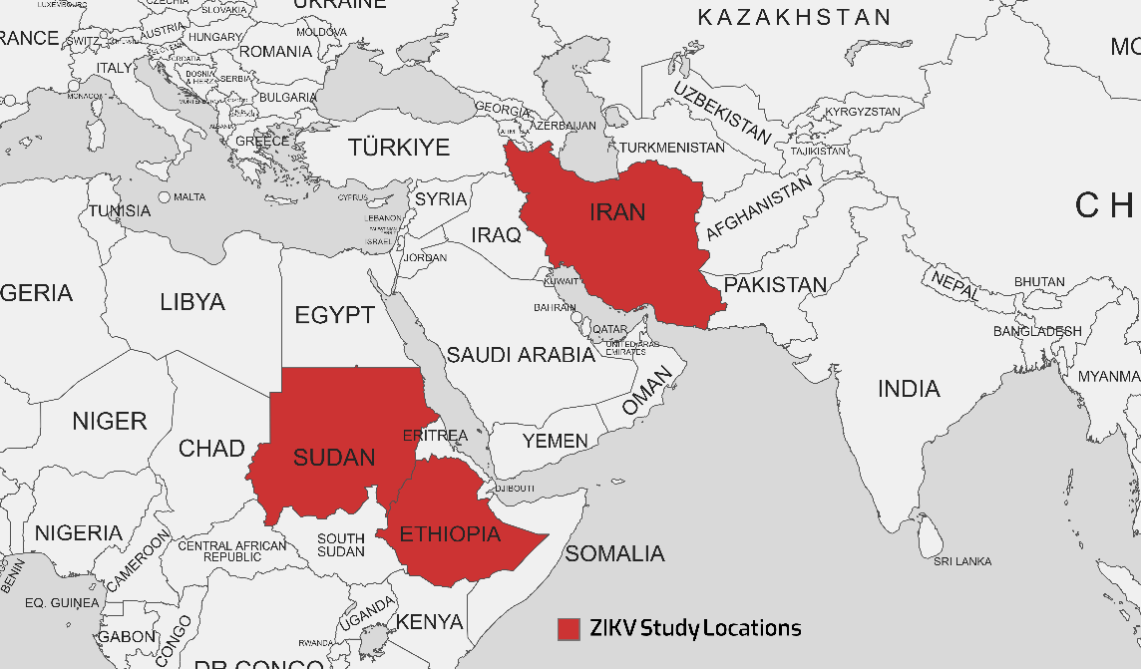
Geographical map of the ZIKV cases distribution as reported in various studies

ZIKV was first isolated from Rhesus monkeys in 1947 and then from the *Aedes* mosquito in 1948 in the Zika forest of Uganda. The first documented case of human infection occurred in Nigeria in 1954. The ZIKV pandemic began in Brazil and spread to 60 different nations in 2015. A significant outbreak was observed in Brazil in 2016 [71], [63], [87]. Additionally, mosquitoes transmit ZIKV within a sylvatic ecosystem during an enzootic cycle that involves non-human primates, specifically monkeys. These infected mosquitoes can spread the disease to humans during an epidemic cycle. People infected with ZIKV often recover spontaneously; thus, they may not require specific treatment. While the demand for safe and effective ZIKV vaccines is global, there are no approved and readily accessible antiviral medications that can effectively prevent or cure the infection [86], [88]. *A. aegypti* and *A. albopictus* mosquitoes are likely Zika vectors in EMRO, given their role in transmission elsewhere. These species’ wide distribution in urban areas where dense populations coexist raises concern for explosive Zika outbreaks, like those seen in the Americas in 2015–2016. Targeting *Aedes* through vector control and personal protection are the first lines of defense against potential Zika emergence [71], [63]. Table 7 (Tab. 7) presents the results of studies conducted on the ZIKV that showed that seroprevalence rates ranged from 0% in two studies in Sudan and Iran in 2018–2019 to as high as 62.7% in Sudan in 2018. The geographical map of the ZIKV case distribution is shown in Figure 6 (Fig. 6).

### Surveillance, prevention and control measures 

Surveillance systems play a crucial role in monitoring the spread of arboviral diseases and detecting outbreaks at an early stage. These systems rely on various methods, including passive case reporting from healthcare facilities, sentinel surveillance through selected health centers or hospitals, epidemiological investigations, and serological tests. Timely reporting of suspected cases allows public health authorities to initiate appropriate control measures, such as vector control activities, vaccination campaigns, etc. [89].

Prevention and control measures for arboviral diseases in the EMRO region involve a multi-faceted approach. These include vector control strategies such as reducing mosquito breeding sites, using insecticide-treated bed nets, and indoor residual spraying. Vaccination programs have also been implemented for certain arboviral diseases, such as dengue fever [130]. Vaccination is now seen as a viable future option by a significant portion of the world's population. Few arboviral vaccinations are available, but they constantly threaten human and animal health [1], [130]. Insect-specific viruses (ISVs) present new potential for vaccine development because they cannot reproduce in vertebrates or their cells [1].

Public health campaigns are regularly conducted to highlight the importance of personal protective measures such as wearing long sleeves and using insect repellents. In addition, healthcare professionals are provided with training programs on early diagnosis and case management. Arboviruses can survive in dormant mosquito eggs for months or even years before the rainy season causes the emergence of healthy but infected mosquito larvae. These viruses frequently take advantage of the longer life cycles of some tick species, surviving for years through the trans-stadial stages and reproducing at low rates [2]. 

## Discussion

This review synthesizes evidence on the epidemiology of major arboviruses in the EMRO between 2014–2023. The data reveals a significant burden of viral diseases across the EMRO region, with varying prevalence rates observed for different pathogens. Notably, the CCHFV exhibits a wide range of prevalence, from 0.4% in Iran (2019) [20] to 64% in the same country (2015) [29], with an overall average of 33%. This highlights the need for robust prevention and control measures to combat CCHFV in the region. 

Furthermore, the data underscores the alarming prevalence of DENV in certain areas, with Pakistan reporting a staggering 100% rate in 2021 [50]. The distribution of DENV serotypes also varies, with serotypes 2 and 3 being the most prevalent. This information is crucial for developing targeted interventions and vaccines against the circulating serotypes [90], [91]. 

WNV antibodies were commonly detected as well, pointing to extensive WNV transmission in the region [70]. RVFV and YFV were detected less frequently; however, the data was limited. Finally, ZIKV was found circulating in Sudan in 2018 at a high rate of 62.7%, indicating active transmission [85].

The significant variability in reported prevalence highlights the need for standardized methods and systematic surveillance within and between EMRO countries. Differences in geographic location, climate, seasons, study population, sample size, and assay techniques likely contributed to the observed ranges. However, clear hot spots of transmission are evident for some viruses like DENV in Pakistan. 

**Figure 7:**
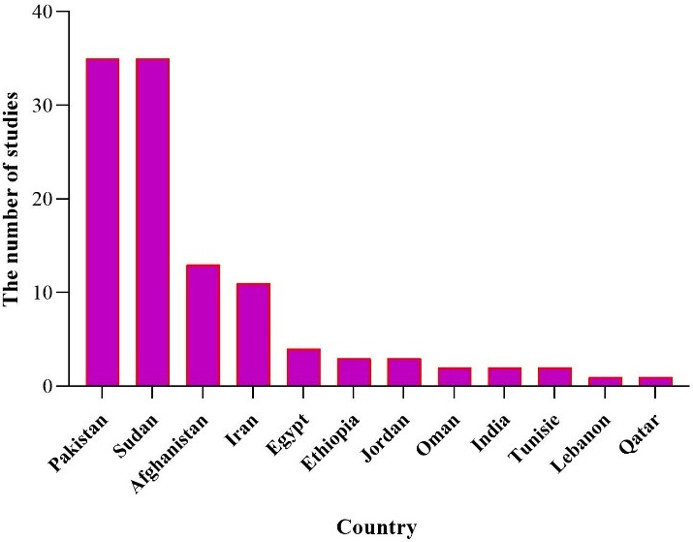
Number of studies conducted in each country during 2014–2024

**Figure 8:**
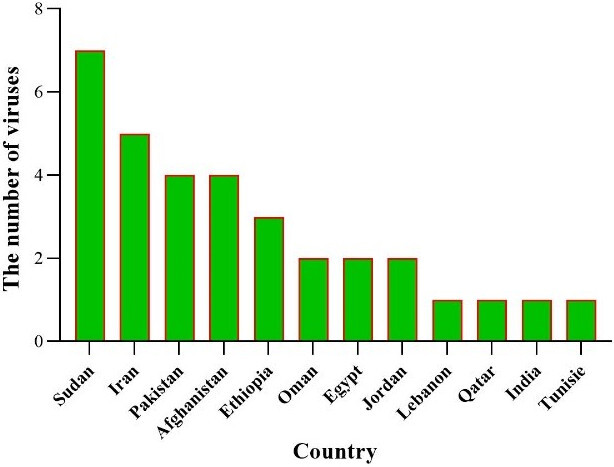
Number of viruses examined in each country during 2014–2024

**Figure 9:**
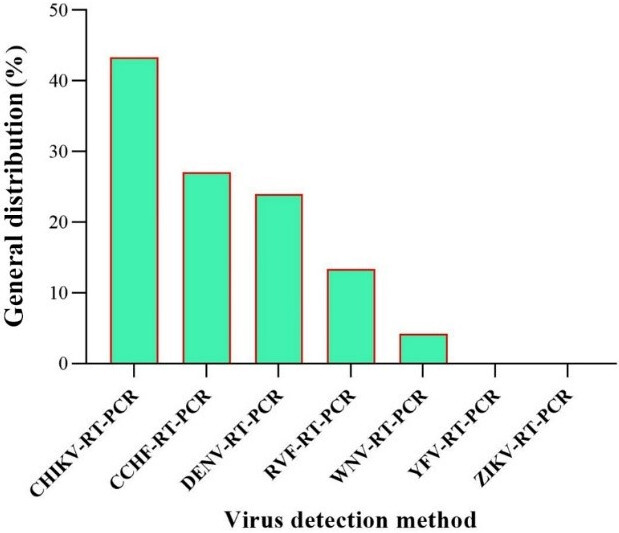
General Distribution of arbovirus detection by RT-PCR methods in the EMRO region

**Figure 10:**
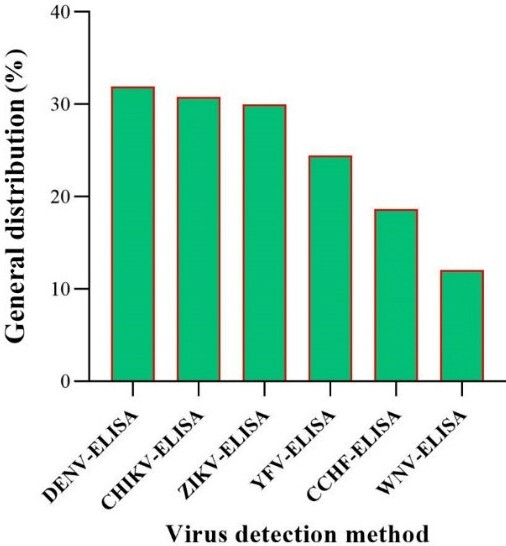
General distribution of arbovirus detection by ELISA methods in the EMRO region

Overall, while this review synthesizes recent evidence on arbovirus epidemiology in EMRO countries, the limitations may restrict the ability to fully characterize transmission patterns and risk factors across the region. Improved coordination and data sharing between regional public health authorities could strengthen the evidence base for targeted prevention and control measures. Given the significance of arboviruses and their proliferation in various regions worldwide, including countries in the EMRO region, there is a pressing need for research and preventative measures to curb their escalating spread. The majority of the detected DENV infections presented were accompanied by bleeding, indicative of dengue hemorrhagic fever. According to WHO guidelines, this is recognized as one of the most severe forms of the disease [39]. To facilitate readers’ understanding, a holistic view of the overall results is depicted in Figure 7 (Fig. 7), Figure 8 (Fig. 8), Figure 9 (Fig. 9), and Figure 10 (Fig. 10). The majority of arbovirus studies were conducted in Pakistan and Sudan, with 35 studies, followed by Iran, with 18 studies. This suggests that Pakistan and Sudan are at the forefront of arbovirus research in the EMRO region. The remaining countries have conducted far fewer studies (Figure 7 (Fig. 7)).

Sudan has examined the most viruses, followed by Iran, Pakistan, and Ethiopia. This suggests that Sudan has tested all seven viruses mentioned in this study within its own borders (Figure 8 (Fig. 8)). 

The most common viruses detected were CHIKV, CCHF, DENV, RVF, WNV, YFV, ZIKV, and CCHF. This suggests that these viruses are of major public health importance in the EMRO region. WNV was 10.75% by ELISA and 2.93% by RT-PCR. In addition, the RT-PCR method failed to detect ZIKV and YFV, which could indicate that the ELISA test is more sensitive than RT-PCR in the case of these viruses (Figure 9 (Fig. 9)).

The highest detection rate is observed for DENV-ELISA at approximately 33%, while the lowest is for WNV-ELISA at about 12%. This figure underscores the varying prevalence of these viruses as detected by ELISA, highlighting the importance of continued monitoring and research (Figure 10 (Fig. 10)).

The EMRO region faces several challenges in addressing arboviral diseases. Limited resources, including funding, trained personnel, and laboratory capacity, can hinder surveillance efforts and timely response to outbreaks. The lack of well-established surveillance systems in some countries within the EMRO may result in underreporting or delayed detection of cases. Climate change is also a factor that can influence the spread of vectors and increase the risk of arboviral diseases [89]. 

### Limitations 

This review has some limitations that should be considered when interpreting the findings. First, we focused solely on evidence of arboviral antibodies or genome presence in human blood/serum samples. Studies detecting arboviruses in non-human hosts or vectors were excluded. Second, we restricted our search to publications from 2014 to January 2024 to examine current epidemiological patterns. Relevant studies before 2014 may have been missed. Third, full-text access was not available for some identified articles, which limited data extraction. Fourth, there was heterogeneity in study design, sampling methods, diagnostic techniques, and reporting formats between the included studies. Finally, the search was restricted to English language publications and thus relevant studies published in other languages may have been overlooked. 

## Conclusions

This study has provided a detailed overview of the prevalence of arboviruses within the EMRO, highlighting the significant public health challenge posed by these pathogens. Our findings indicate that arboviruses are increasingly becoming a cause for concern in this region due to factors such as climate change, urbanization, and increased human mobility. The data presented underscores the urgent need for enhanced surveillance systems to monitor arbovirus activity and the vectors responsible for their transmission. It is imperative that member states in the EMRO region collaborate to strengthen vector control measures and develop strategies to mitigate the risk of outbreaks. Public health initiatives must also focus on community education to raise awareness about the prevention of arboviral diseases. Furthermore, our research calls for the integration of arbovirus monitoring into national health systems, ensuring that resources are allocated efficiently to combat the spread of these diseases. The establishment of regional centers of excellence for arbovirus research can facilitate the sharing of knowledge and technical expertise, fostering a proactive approach to disease management and control.

In conclusion, the threat of arboviruses in the EMRO region is a multifaceted issue that requires a coordinated, multidisciplinary response. By adopting a holistic approach that encompasses surveillance, prevention, and research, the EMRO region can effectively address the challenges posed by arboviruses and safeguard the health of its populations.

## Notes

### Competing interests

The authors declare that they have no competing interests.

### Authors’ ORCID

- Owliaee I: https://orcid.org/0000-0002-9695-4938
- Khaledian M: https://orcid.org/0000-0002-8123-7856
- Shojaeian A: https://orcid.org/0000-0002-1166-385X
- Jalilian FA: https://orcid.org/0000-0003-3134-1441

### Funding

None. 

## Figures and Tables

**Table 1:**
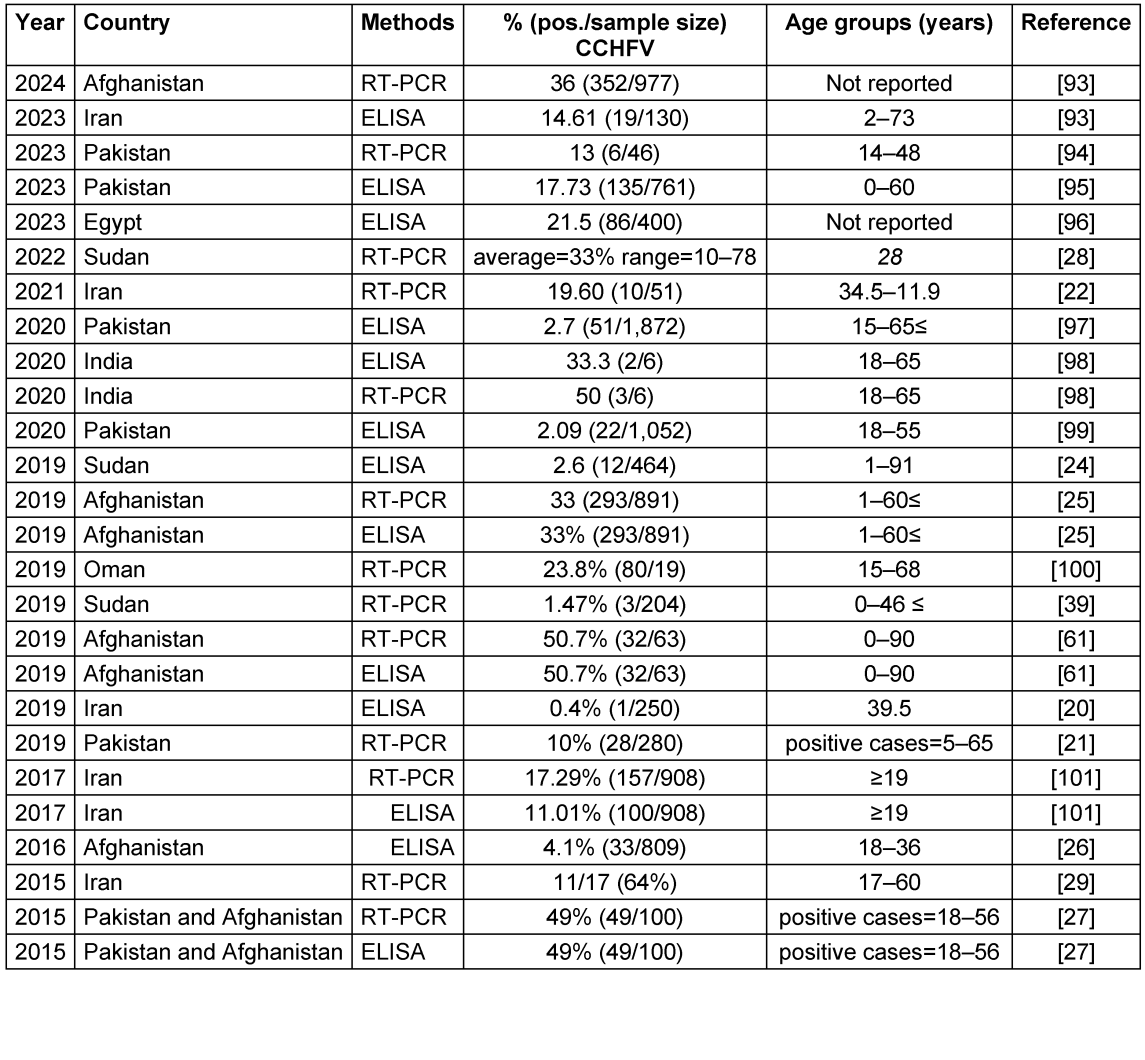
Summary of the CCHFV seroprevalence studies in the EMRO region, 2015–January 2024

**Table 2:**
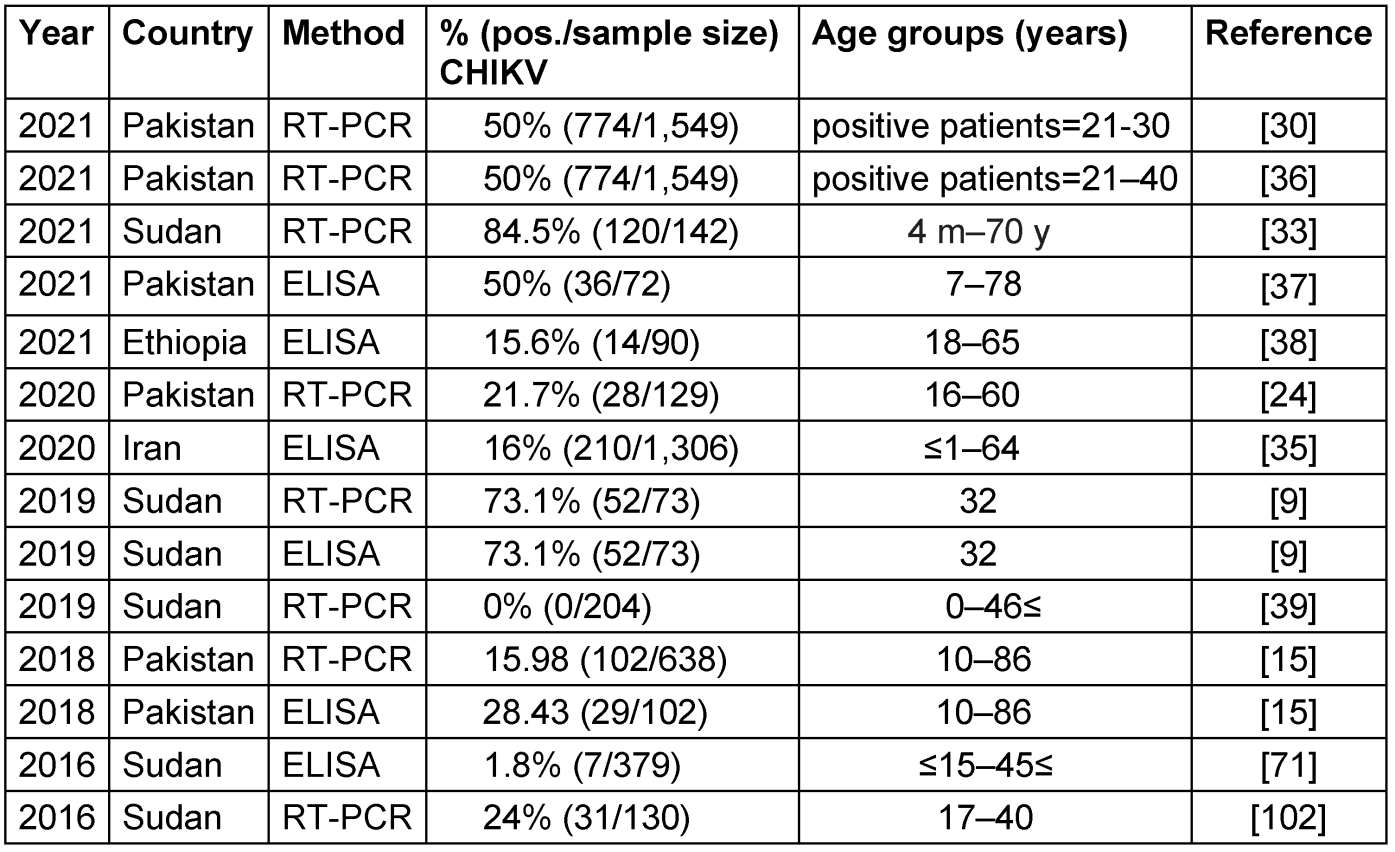
Summary of the CHIKV seroprevalence studies in the EMRO region, 2016–2021

**Table 3:**
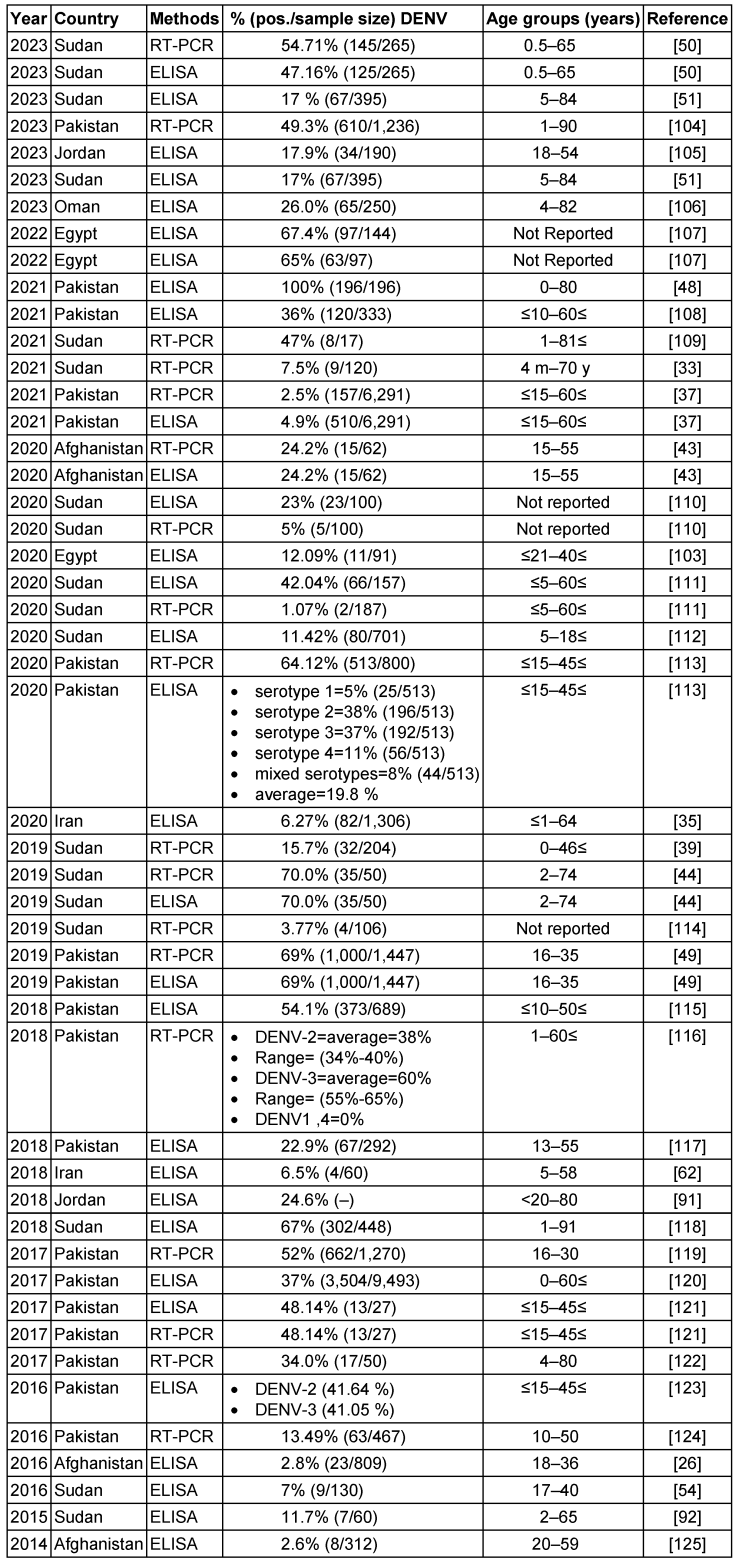
Summary of the DENV seroprevalence studies in the EMRO region, 2014–2023

**Table 4:**
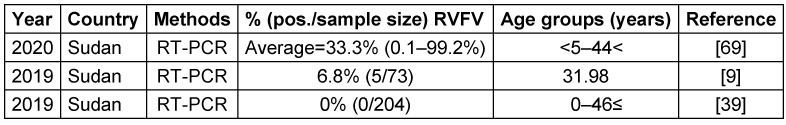
Summary of the RVFV seroprevalence studies in the EMRO region, 2019–2020

**Table 5:**
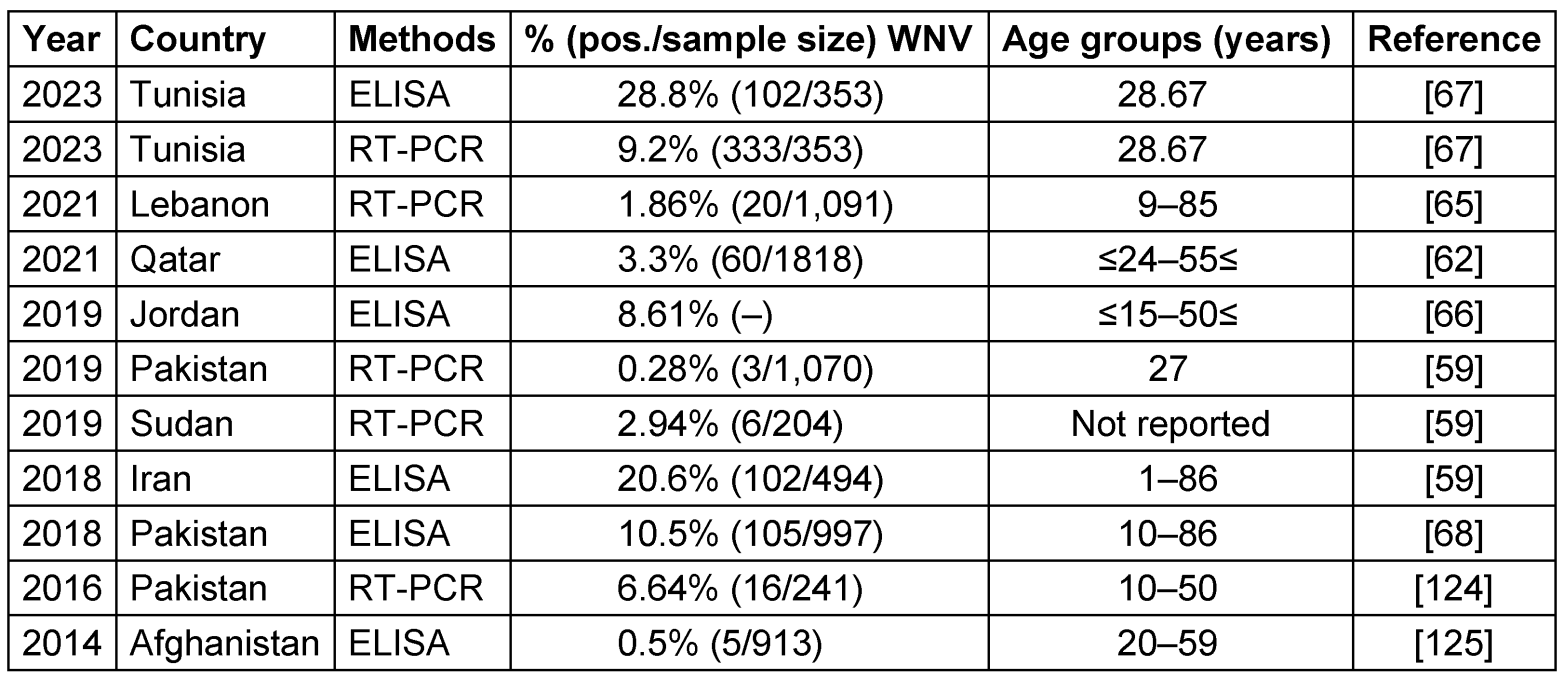
Summary of the WNV seroprevalence studies in the EMRO region, 2014–2023

**Table 6:**
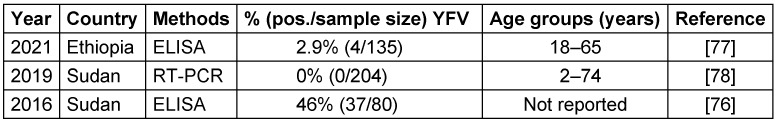
Summary of the YFV seroprevalence studies in the EMRO region, 2016–2021

**Table 7:**
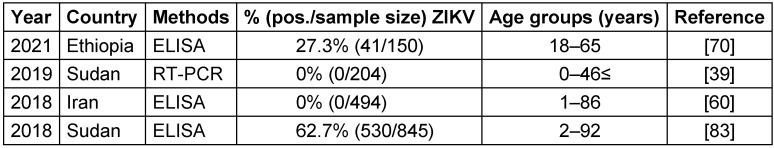
Summary of the ZIKV seroprevalence studies in the EMRO region, 2018–2021
